# Supplementary material for: WordCommentsAnalyzer: A windows software tool for qualitative research
Source: F1000Res. 2018 Sep 4;7:536. Originally published 2018 May 3. [Version 2] doi: 10.12688/f1000research.14819.2 (PMC6137411; doi:10.12688/f1000research.14819.2)
Supplement: Supplementary file 3 [file f1000research-7-17614-s0002.tgz › b2a79496-4ada-47c4-9682-a99ce867836d.zip!/Eight Iranian Health Journals/Journal of Research in Rehabilitation Sciences _27.docx]

© Journal of Research in Rehabilitation Sciences . This work is licensed under CC BY-NC 4.0 (https://creativecommons.org/licenses/by-nc/4.0/).

Volume & Issue: دوره 9، شماره 6: 1392

Title: تاثیر پوسچر پرونیتوری پا و ارتز گوه داخلی پاشنه و کف پایی بر بالانس استاتیک در سالمندان

http://jrrs.mui.ac.ir/index.php/jrrs/article/view/1507

چكيده  تاثیر پوسچر پرونیتوری پا و ارتز گوه داخلی پاشنه و کف پایی بر بالانس استاتیک در سالمندان  مقدمه: افزایش پرونیشن پا در سالمندان گزارش شده است. این امر می‌تواند باعث تغییر در شکل پا و حرکت بیش از حد مفاصل پا شود که ممکن است بر تعادل تاثیر بگذارد. مداخلات ارتزی برای بهبود پوسچر پا ممکن است تاثیرات مهمی بر تعادل سالمندان داشته باشد. بنابراین هدف از این مطالعه بررسی تاثیر پوسچر پرونیتوری پا و ارتز گوه داخلی پاشنه و کف پایی بر بالانس استاتیک در افراد سالمند است. مواد و روش‌ها: 9 سالمند سالم با پوسچر پرونیتوری پا (میانگین سنی5/5 ±1/67 سال) و 14سالمند سالم با پوسچر نرمال پا (میانگین سنی 9/5 ± 1/67 سال) مورد مطالعه قرار گرفتند. از شاخص پوسچر پا برای تعیین پوسچر پا استفاده شد ) میانگین شاخص پوسچر پا در گروه پرونیتوری (0.7± 7.5 )، میانگین شاخص پوسچر پا در گروه نرمال (1.4± 3.8 ) . بالانس استاتیک طی ایستادن روی دو اندام با چشمان باز و بسته و با پوشیدن کفش با استفاده از دستگاه سکوی نیرو در چهار وضعیت مختلف (دو نوع ارتز گوه داخلی پاشنه و کف پایی 5 و 8 درجه عرضی و دو نوع کفی ساده تمام طول هم ارتفاع با گوه‌ها) ارزیابی شد. هر وضعیت سه بار تکرار می‌شد. بالانس استاتیک به وسیله پارامترهای متوسط مسافت طی شده مرکز فشار در راستای قدامی خلفی و داخلی خارجی و مقدار برآیند آن، مجموع مسافت طی شده مرکز فشار در راستای قدامی خلفی و داخلی خارجی و مقدار برآیند آن، متوسط سرعت مرکز فشار در راستای قدامی خلفی و داخلی خارجی و مقدار برآیند آن و مساحت ناحیه بیضی محاط بر محدوده حرکت مرکز فشار، اندازه‌گیری شد. یافته‌ها: تفاوت معنی‌داری در متغیرهای بالانس استاتیک در دو گروه سالمند با پوسچر نرمال و پرونیتوری پا با وجود کفی ساده وجود نداشت (0.05P>)، اما افراد با پای پرونیتوری مقادیر میانگین بالاتری در متغیرهای بالانس استاتیک نسبت به افراد با پاهای نرمال داشتند. استفاده از ارتز در هیچ یک از دو گروه تاثیر معنی‌داری نداشت (05.0P>). وضعیت چشم بسته منجر به افزایش معنی‌دار برایند کل مسافت طی شده مرکز فشار، مجموع مسافت طی شده مرکز فشار و متوسط سرعت مرکز فشار در راستای داخلی خارجی و مساحت ناحیه بیضی محاط بر محدوده حرکت مرکز فشار گردید. همچنین اثر متقابل ارتز، پوسچر پا و وضعیت بینایی معنی‌دار  نبود(05/0P>).  نتیجه‌گیری: هر چند تفاوت معنی‌داری در پارامترهای بالانس بین دو گروه مشاهده نشد، اما افراد با پای پرونیتوری مقادیر میانگین بالاتری در اغلب پارامترهای بالانس نسبت به گروه نرمال داشتند که شاید نشانگر اختلال تعادل در سالمندان با پای پرونیتوری باشد. ارتز گوه داخلی تاثیری بر بالانس افراد با پای پرونیتوری نداشت، بنابراین می‌توان از آن برای بهبود عملکرد پا و مچ پا بدون ترس از اختلال در بالانس استفاده کرد.

Title: بررسی تأثیر مواجهه با مخلوط حلال های آلی بر پردازش شنوایی مرکزی

http://jrrs.mui.ac.ir/index.php/jrrs/article/view/1328

چکیده مقدمه: حلال‌ها عموماً به صورت بخارشده استنشاق می‌شوند و از طریق مجرای تنفسی جذب می‌شوند. مطالعات مختلفی آسیب مرکزی شنوایی ناشی از مواجهه با حلال‌ها را گزارش کرده‌اند. با توجه به نوروتوکسیسیتی احتمالی ناشی از مواجهه با حلال‌ها، پردازش شنوایی مرکزی ممکن است در افرادی که در معرض این حلال‌ها قرار دارند متأثر شود و این ممکن است بطور نامطلوبی بر عملکرد افراد در شرایط شنیداری روزمره تأثیر گذارد. هدف از این مطالعه بررسی پردازش شنوایی مرکزی در افراد در معرض حلال‌های آلی بود. مواد و روش‌ها: این مطالعه مقطعی بر روی 62 فرد در معرض حلال و 93 فرد بدون مواجهه شاغل در یک کارخانه خودروسازی انجام شد. همه افراد دارای آستانه شنوایی و تیمپانوگرام و رفلکس اکوستیک و زوال رفلکس اکوستیک طبیعی بودند. پس از انجام آزمون اعداد دایکوتیک نتایج درصد پاسخ‌های صحیح افراد استخراج شد و با استفاده از آزمون ناپارامتری Mann-Witney U   مورد تجزیه و تحلیل قرار گرفت. یافته‌ها: در آزمون اعداد دایکوتیک گوش چپ و گوش راست بین گروه مواجهه با حلال و گروه بدون مواجهه تفاوت معنی‌داری معنی‌داری پیدا نشد، ولی برای امتیاز کلی اعداد دایکوتیک تفاوت معنی‌داری معنی‌داری پیدا شد. نتیجه‌گیری: عملکرد افراد در معرض حلال نسبت به افراد بدون مواجهه ضعیف‌تر بود که دلالت بر وجود مشکل در جنبه‌های تلفیق و تفکیک دوگوشی این افراد می‌کند.

Title: تأثیر آموزش مهارت‌های اجتماعی بر بهبود عزت نفس نوجوانان با کم‌توانی ذهنی

http://jrrs.mui.ac.ir/index.php/jrrs/article/view/911

چكيده      مقدمه: عزت نفس نقش اساسی در همه ابعاد زندگی افراد به ویژه نوجوانان با کم‌توانی ذهنی دارد. بنابراین بر آموزش برنامه‌هایی که منجر بهبود عزت نفس می‌شود تأکید شده است. هدف این پژوهش بررسی اثربخشی آموزش مهارت‌های اجتماعی بر بهبود عزت نفس نوجوانان با کم‌توانی ذهنی در استان مازندران بود. مواد و روش‌ها: در مطالعه نیمه تجربی حاضر با طرح پیش آزمون-پس آزمون با گروه کنترل، با استفاده از روش نمونه‌گیری تصادفی خوشه‌ای چند مرحله‌ای 40 نوجوان دختر با کم‌توانی ذهنی 14 تا 18 ساله انتخاب و به طور تصادفی در یک گروه آزمایش و یک گروه كنترل جایگزین شدند، به طوری که هر گروه 20 نفر بود. به گروه‌ آزمایش مهارت‌های اجتماعی در 8 جلسه 90 دقیقه‌ای آموزش داده شد ولی به گروه كنترل این نوع آموزش‌ ارائه نشد. همه آزمودنی‌ها در مرحله پیش‌آزمون و پس‌آزمون با استفاده از پرسش‌نامه عزت نفس کوپر اسمیت ارزیابی شدند. اطلاعات جمع‌آوری شده با استفاده از تحلیل کواریانس چندمتغيري، در نرم‌افزار SPSS نسخه 16 (version 16, SPSS Inc., Chicago, IL) تحلیل شد. یافته‌ها: نتایج کلی تحلیل کواریانس چندمتغیری برای خرده مقیاس‌های عزت نفس نشان داد که گروه آزمایش و کنترل حداقل در یکی از چهار خرده مقیاس عزت نفس خود، عزت نفس اجتماعی، عزت نفس خانواده یا عزت نفس تحصیلی تفاوت معنی‌داری دارند (0027/0P=). نتایج نهایی تحلیل کواریانس چندمتغیری نشان داد که آموزش مهارت‌های اجتماعی بر عزت نفس خود (0033/0P=)، عزت نفس اجتماعی (0041/0P=)، عزت نفس خانواده (0039/0P=) و عزت نفس تحصیلی (0026/0P=) نوجوانان با کم‌توانی ذهنی اثر مثبت و معنی‌داری داشت. نتیجه‌گیری: برنامه آموزشی مهارت‌های اجتماعی منجر به بهبود عزت نفس و خرده مقیاس‌های آن در نوجوانان با کم‌توانی ذهنی شد. بنابراین، برنامه‌ریزی جهت آموزش مهارت‌های اجتماعی به آنها اهمیت ویژه‌ای دارد.

Title: بررسی تعادل ایستایی در بیماران مبتلا به اختلالات عصبی و اسکلتی عضلانی با استفاده از رویکردهای خطی و غیرخطی

http://jrrs.mui.ac.ir/index.php/jrrs/article/view/1169

چکیده مقدمه: تعادل (Stability) عبارتست از توانایی فرد در حفظ حالت ایستاده که از طریق استراتژی‌های ترکیبی سیستم‌های اسکلتی عضلانی مختلف تسهیل می‌شود. به منظور ارزیابی توانایی فرد در حفظ ثبات ایستادن روش‌های مختلفی از جمله روش خطی و روش غیرخطی به کار می‌رود. مشخص نگردیده است که کدام روش در نشان دادن ثبات وضعیت (Posture) افراد با اختلالات عصبی و اسکلتی عضلانی مختلف قابلیت بیشتری دارد. از این رو هدف از مطالعه حاضر بررسی ثبات افراد مبتلا به ضایعات عصبی و اسکلتی عضلانی مختلف با رویکرد خطی و غیرخطی است. مواد و روش‌ها: چهار گروه از افراد سالم، دارای اختلال صافی کف پا، پارکینسون و پرتس در این مطالعه وارد شدند. جهت ارزیابی ثبات از دستگاه سکوی اندازه‌گیری نیروی کیستلر استفاده شد. تفاوت پارامترهای خطی (دامنه، سرعت و طول مسیر مرکز فشار) و غیرخطی (آنتروپی تقریبی مرکز فشار) با تست two sample t test آزمون گردید.  یافته‌ها: میانگین سرعت مرکز فشار در جهت داخلی/خارجی در افراد دارای صافی کف پا و مبتلایان پرتس به ترتیب 6./405±5/1621 و 195±4/878 میلی­متر بر دقیقه بود (p≤0/05). میانگین آنتروپی تقریبی در جهت قدامی/خلفی در افراد دارای صافی کف پا و مبتلایان پرتس به ترتیب  16/0±94/0و 21/0±99/0 بود در حالی‌که این پارامتر در بیماران پارکینسون 06/0±45/0 بود. تفاوت میانگین آنتروپی تقریبی در جهت قدامی/خلفی گروه سالم با تمام گروه‌ها معنی‌دار بود (p≤0/05). نتیجه‌گیری: به نظر می‌رسد رویکرد غیرخطی در نشان دادن تفاوت ثبات افراد با اختلالات عصبی و اسکلتی عضلانی مختلف حساسیت بیشتری دارد. البته توصیه می‌شود در صورت انتخاب روش خطی جهت آنالیز اطلاعات، بجای تنها یک پارامتر از چند پارامتر استفاده گردد. افزایش یا کاهش آنتروپی تقریبی وابسته به نوع ضایعه موقعیت‌های بی‌ثباتی را گزارش می‌کند.

Title: تأثیر یادگیری کم خطا و پرخطا بر متغیرهای کینماتیکی اجرا در یک تکلیف پرتابی: یک مطالعه مقدماتی

http://jrrs.mui.ac.ir/index.php/jrrs/article/view/885

چکیده مقدمه: بررسی تفاوت اثر یادگیری کم خطا و پرخطا بر متغیرهای کینماتیکی اجرا. مواد وروش‌ها: 24 دانشجوی دختر در سه گروه کم‌خطا، پرخطا و ثابت قرار گرفتند. به گروه‌ها دستورالعمل نحوه پرتاب داده نشد. شرکت‌کنندگان گروه پرخطا به ترتیب از فواصل 5/3، 25/3، 3، 75/2، 5/2 متری، گروه کم‌خطا عکس این فواصل و گروه ثابت، از فاصله 3 متری از هدف به تمرین پرداختند. پس از سه روز تمرینی آزمون‌های اکتساب، یادداری و ...  انجام شد. از دستگاه تجزیه و تحلیل حرکت برای جمع‌آوری داده‌های کینماتیک در تمام مراحل استفاده شد. یافته‌ها: در آزمون یادداری، عملکرد پرتاب گروه کم‌خطا به طور معنی‌داری بهتر از دو گروه دیگر بود و در آزمون انتقال گروه پرخطا دچار افت شد. در اجرای تکلیف ثانویه شناختی بین دو گروه کم خطا و ثابت تفاوت معنی‌داری یافت نشد. در تحلیل متغیرهای کینماتیکی گروه کم خطا به الگوی ماهرانه تکلیف حرکتی مورد نظر نزدیک شد ولی گروه پرخطا فاصله زیادی تا اجرای الگوی ماهرانه تکلیف داشت. نتیجه‌گیری: یادگیری کم خطا روشی کارآمد در یادگیری مهارت پرتابی تحقیق حاضر است.

Title: بررسی نیازهای آموزشی توانبخشی مادران کودکان اتستیک

http://jrrs.mui.ac.ir/index.php/jrrs/article/view/864

چکیده مقدمه: اُتيسم اختلال عصب شناختي رو به گسترشي است كه ابعاد مختلف عملكرد كودك و خانواده را تحت تاثير قرار مي‌دهد و نيازمند ارايه خدمات توانبخشي و درماني چند بعدي و گاه مادام‌العمر است لذا پژوهش حاضر با هدف بررسی نیازهای مادران کودکان اتیستیک انجام شده است. مواد و روش‌ها:این پژوهش از نوع نیازسنجی است. گروه نمونه، والدین 41 نفر از کودکان اتیسم را شامل می‌شود که به صورت نمونه‌گیری در دسترس از سه کلینیک اتیسم شهرستان مشهد، انتخاب شدند. شرکت‌کنندگان، پرسش‌نامه پژوهشگرساخته، شامل نُه مقوله آموزشی- توانبخشی را کامل کردند. پایایی کل پرسش‌نامه (نُه مقوله) به شیوه ضریب آلفای کرونباخ 94/0= a محاسبه شد. يافته‌ها: نتایج تی تک نمونه‌ای نشان داد که والدین کودکان اتیسم، همه مقوله‌های آموزشی- توانبخشی را مورد نیاز ارزیابی کردند و چهار مقوله آشنایی با مسایل خودیاری کودک، آشنایی با مهارت‌های بازی کودک و بازی کردن والدین با کودک، آشنایی با مشکلات شناختی و ذهنی کودک و آموزش مهارت‌های ارتباطی را بسیار مورد نیاز ارزیابی کردند. همچنین، نتایج تحلیل واریانس چند متغیره نشان داد که سن کودک، ترتیب تولد کودک، سن مادر و میزان تحصیلات والدین، در اولویت بندی نیازها، تأثیری نداشته است. نتيجه‌گيري: به كارگيري نتايج و اولويت‌هاي مستخرج از نيازسنجي حاضر مي‌تواند متخصصان و درمانگران را جهت ارايه خدمات توان‌بخشي و روانشناختي از اولويت بالا يا در حد بسيار مورد نياز تا اولويت‌هاي بعدي يا در حد مورد نياز ياري رسانند. .

Title: بررسی ارتباط درد وترس از درد با ناتوانی و متغیرهای وضعیتی در بیماران مبتلا به کمر درد مزمن غیراختصاصی

http://jrrs.mui.ac.ir/index.php/jrrs/article/view/1180

چکیده مقدمه: ترس از درد یکی از عوامل روانی مهم در ناتوانی و غیبت از کار در بیماران مبتلا به کمردرد مزمن می‌باشد. هدف این مطالعه بررسی ارتباط ترس از درد با پارامترهای ثبات پوسچرال در بیماران کمردرد مزمن غیر اختصاصی می‌باشد. مواد و روش‌ها: در این مطالعه نیمه‌تجربی، 51 بیمار مبتلا به کمردرد مزمن غیراختصاصی بررسی شدند. شدت درد، ترس از درد و ناتوانی با پرسش‌نامه و پارامترهای ثبات پوسچر (مقدار ، فرکانس و سرعت جابجایی مرکز فشار) با صفحه نیرو اندازه‌گیری شد. تحمل عضلات شکم و کمر به ترتیب با آزمون‌های sit up و surensen اندازه‌گیری شد. ارتباط شدت درد و ترس از درد با پارامترهای ثبات پوسچر با در نظر گرفتن اثر سن، شاخص توده بدنی، فعالیت فیزیکی، تحمل عضلات شکم و کمر و جنس با آزمون multivariate general linear modle تحلیل شد. یافته‌ها : در حالت ایستادن روی یک پا، درد و ترس از درد (فیزیکی) با جابجایی و سرعت جابجایی مرکز فشار در هر دو صفحه قدامی خلفی و داخلی خارجی ارتباط معنی‌داری داشت (01/0p<). همچنین ترس از درد (فیزیکی) با شدت درد و تحمل عضلات کمر ارتباط معنی‌دار داشت(01/0p<). درد و ترس از درد با هیچ کدام از پارامترهای ثبات پوسچر در حالت ایستادن روی دو پا ارتباط معنی‌داری نداشت. نتیجه‌گیری: وجود ارتباط بین ترس از درد و شدت درد با جابجایی و سرعت جابجایی مرکز فشار در حالت ایستادن روی یک پا می‌تواند در به کارگیری استراتژی متفاوت و ساده‌تری در ارزیابی و درمان بیماران مبتلا به کمردرد مزمن کمک‌کننده باشد که شامل توجه به وضعیت روحی روانی اجتماعی آن‌ها از جمله میزان ترس از درد آن‌ها باشد.

Title: بررسي برخي شاخص هاي آكوستيكي صوت کودکان مبتلا به کم شنوايي شديد تا عميق 6 تا 11 سال

http://jrrs.mui.ac.ir/index.php/jrrs/article/view/1224

چکيده مقدمه: عامل اساسي تأثيرگذار بر توليد صداي انسان شنوايي است. برخورداري از سيستم شنوايي سالم و نيز فيدبک شنيداري هنجار مي‌تواند جنبه‌هاي مهمي از گفتار شامل صوت، و توليد آواهاي گفتاري را کنترل کرده و فرد از اين طريق قادر است اشتباهات خود را تصحيح کند. هنجار بودن شاخص‌های صوتی تا حد زيادي به عملکرد سيستم شنيداري وابسته است. مواد و روش‌ها: اين مطالعه به صورت مقطعي- مقايسه‌اي روي27کودک کم شنواي شديد تا عميق و 27 کودک با شنوايي هنجار در محدوده سني6 تا 11 سال كه از نظر سن و جنس همسان بودند، به روش نمونه‌گيري غير احتمالي در مدرسه باغچه‌بان و مدارس منطقه 2 تهران انجام شد. پس از انجام آزمون‌هاي شنوايي، شاخص‌هاي صوتی كودكان شامل فركانس پايه، تغييرات فركانس، تغييرات شدت، آشفتگي در زير و بمي صوت، آشفتگي در شدت صوت و نسبت نويز به هارمونيك آناليز شد. كليه داده‌هاي كمّي حاصله با استفاده از نرم‌افزار SPSS و آزمون‌هاي t-test و Pearson Correlation انجام شدند. يافته‌ها: در کودکان کم‌شنوا شاخص‌هاي فرکانس پايه صوت، محدوده بالاي فرکانس پايه و تغييرات در دامنه شدت صوت بيش از گروه هنجار بود و تفاوت معنی‌داري يافت گرديد. با اينکه شاخص‌هاي آشفتگي در فركانس و شدت صوت، درصد تغييرات فرکانس پايه و نسبت نويز به ‌هارمونيك صوت کودکان کم‌شنوا از کودکان با شنوايي هنجار بيشتر بود تفاوت معنی‌داري مشاهده نشد. بين متغير آشفتگي در فرکانس پايه صوت و فاصله بين آستانه‌هاي فرکانس 500 هرتز تا 2000 هرتز همبستگي معني‌داري يافت شد. نتيجه‌گيري: نقص در سيستم شنوايي در سطح شديد تا عميق به دليل کاهش فيدبک مناسب صوتي بر شاخص‌هاي آکوستيکي صوت کودکان تأثير مي‌گذارد، مهم‌ترين شاخص‌هايي که دچار تغيير مي‌شوند فرکانس پايه صوت و دامنه شدت صوت مي‌باشد.

Title: تأثیر نوع کفش بر متغیرهای مرتبط با آسیب استرس فراکچر ساق حین دویدن

http://jrrs.mui.ac.ir/index.php/jrrs/article/view/1094

چکیده مقدمه: مطالعات نشان داده‌اند که شکستگی استرسی درشت نی یکی از شایع‌ترین آسیب‌ها در میان افرادی است که به طور تفریحی و منظم به ورزش دو مشغول می‌باشند. هدف از مطالعه حاضر بررسی تأثیر دو نوع کفشمختلف بر متغیر‌های مرتبط با شکستگی استرسی درشت نی حین دویدن بود. مواد و روش ها: در این مطالعه نیمه تجربی، 25مرد جوان سالم(با میانگین سنی 27/2±21 سال، قد 39/5±93/176 سانتی‌متر و جرم 84/8±30/72 کیلوگرم)، در شرایط کفش ناپایدار، کفش کنترل و پابرهنه دویدند و متغیرهای وابسته به آسیب شکستگی استرسی درشت نی شامل حداکثر نیروهای عمودیفعال و غیرفعال و همچنین سرعت بارگذاری نیروی عمودیبا استفاده ازصفحه نیرواندازه گیری و مقایسه شد. یافته‌ها: سرعت بارگذاری نیروی عمودی 80% در کفش ناپایدار به طور معنی داری بیشتر از کفش کنترل بود(001/0P=). حداکثر نیروی عمودی غیرفعال در کفش ناپایدار در مقایسه با کفش کنترل (000/0=P) و وضعیت پابرهنه به طور معناداری افزایش یافت (039/0=P). با وجوداین، سرعت بارگذاری نیروی عمودی و حداکثر نیروی عمودی غیرفعال در کفش کنترل به طور معنی‌داری کمتر از پای برهنه بود(به ترتیب001/0=P و 002/0=P). نتیجه‌گیری: استفاده از کفش ناپایدار ممکن است دوندگان تفریحی را در معرض خطرات آسیب شکستگی استرسی درشت نی قرار دهد.

Title: بررسي ويژگي‌هاي روان سنجي پرسش‌نامه سنجش اختلالات صدا، ويژه معلمان مقطع ابتدايي شهر اصفهان

http://jrrs.mui.ac.ir/index.php/jrrs/article/view/1337

چکيده مقدمه: معلمان بزرگترين کاربران حرفه‌اي صوت هستند که شيوع اختلالات صوت در آن‌ها حدود 60 درصد مي‌باشد. با توجه به اينکه عمده علائم صوتي معلمان داراي اختلالات صوت، ناشي از تاثيرات شغلي است، طراحي و تدوين پرسش‌نامه‌اي براي سنجش اختلالات صوت در معلمان به منظور تشخيص سريع و کاهش هزينه‌هاي اجتماعي و اقتصادي مي‌تواند از ضروريات باشد. پروتکلي تحت عنوان «پرسش‌نامه سنجش اختلالات صوت ويژه معلمان مقاطع ابتدايي شهر اصفهان» توسط مجيري و اعظميان در سال 1389 طراحي شده‌است که نقطه ضعف آن، عدم تعيين ويژگي‌هاي کاربردي اين ابزار مي‌باشد. لذا هدف پژوهش حاضر تعيين ويژگي‌هاي روان‌سنجي اين پرسش‌نامه به منظور غربالگري سريع اختلالات صوت در اين جمعيت مي‌باشد. مواد و روش‌ها: 52 معلم با صداي نرمال و 52 معلم داراي اختلالات صوت در قالب يک مطالعه توصيفي–تحليلي به شيوه نمونه‌گيري آسان وارد مطالعه شدند. نمونه صوتي از معلمان به منظور ارزيابي اکوستيک و ادراکي صورت گرفت. فرم تعيين شکايت صوتي و پرسش‌نامه توسط معلمان پر شد. از آزمون منحني راک به منظور تعيين حساسيت و ويژگي و نقطه برش پرسش‌نامه استفاده شد. یافته‌ها: در اين پژوهش براي نقطه برش 9، حساسيت 78/0، ويژگي 94/0، ارزش اخباري مثبت 93%، ارزش اخباري منفي81% و نسبت درست‌نمايي 13% بدست آمد. نتيجه‌گيري:  پرسش‌نامه از توان بالايي به منظور تعيين معلمان داراي اختلالات صوت و معلمان با صوت نرمال برخوردار است و مي‌تواند پروتکل مناسبي براي غربالگري اختلالات صوت در معلمان باشد.

Title: تاثیر تمرینات ریباند بر تعادل ایستا و پویای کودکان کم توان ذهنی آموزش‌پذیر

http://jrrs.mui.ac.ir/index.php/jrrs/article/view/1184

چکیده   مقدمه: كودكان كم‌توان‌ ذهني از جمله افرادي هستند كه به علت شرايط ذهني و رواني ويژه در مقايسه با افراد سالم تحرك کمتری داشته و به همین علت دچار ضعف‌هاي جسماني- حركتي مي‌شوند. هدف از اين پژوهش مطالعه تاثیر هشت هفته تمرینات ریباند بر تعادل ایستا و پویای کودکان کم توان ذهنی آموزش‌پذیر بود. مواد و روش‌ها: روش مطالعه نیمه تجربی و تعداد 30 نفر از دانش آموزان پسر 7 تا 11 ساله مدارس استثنایی شهرستان لردگان با میانگین سنی (27/1±46/8) و بهره هوشی 50 تا 70 (88/5±10/62) به صورت هدفمند و در دسترس انتخاب و بر اساس هوشبهر به طور تصادفي جفت شده در دو گروه 15 نفري كنترل و تجربي قرار گرفتند. به منظور ارزیابی تعادل ایستا از زیر آزمون تعادلی قابلیت حرکتی بروئینینکس- اوزرتسکی استفاده شد. گروه تجربی به مدت 8 هفته، هفته‌اي سه جلسه 45 دقیقه‌ای تمرینات ریباند را انجام دادند. همچنین در پایان دوره پس آزمون به عمل آمد. از تحلیل واریانس برای اندازه‌گیری‌های مکرر استفاده شد و سطح معنی‌داری 05/0 محاسبه شد.  يافته‌ها: نتایج نشان داد که میانگین رکورد آزمون تعادل ایستا در پیش آزمون در دو گروه به ترتیب 44/5 و86/6 ثانیه بود و در پس آزمون این میزان به ترتیب به 92/6 و 15/7  ثانیه رسید. همچنین میانگین تعادل پویا در پیش آزمون در دو گروه به ترتیب 42/3 و 37/4 گام بود که در پس آزمون این میزان به ترتیب به 64/4 و 28/4 گام رسید. یافته‌ها بیانگر مثبت تاثیر تمرینات ریباند بر تعادل ایستا (002/0=P) و پویای (001/0=P) کودکان کم‌توان ذهنی بود. نتيجه‌گيري: با توجه به نتایج به دست آمده می‌توان گفت تمرینات ریباند در توان‌بخشي کودکان کم توان ذهنی مؤثر می‌باشد و از این روش می‌توان در توانبخشی حرکتی این کودکان استفاده نمود.

Title: تعیین شاخص قوس کف پا در مقاطع سنی مختلف زنان و مردان شهر اصفهان

http://jrrs.mui.ac.ir/index.php/jrrs/article/view/1070

چكيده                                                                                             مقدمه: با وجود تحقيقات فراوان در خصوص ناهنجاری های کف پا (کف پای صاف و گود) هنوز در باره ی حدود و دامنه طبيعي آنها توافق عمومي وجود ندارد. لذا تحقیق حاضر با هدف تعیین شاخص قوس کف پا در مقاطع سنی مختلف زنان و مردان شهر اصفهان صورت گرفته است. مواد و روش⁯ها: جامعه آماری این تحقیق شامل افراد شهر اصفهان بودند که 250 زن و 250 مرد به صورت تصادفی از شمال، جنوب، شرق، غرب و مرکز شهر اصفهان از بین افراد سالم در 5 مقطع سنی به عنوان نمونه انتخاب و از طریق ارزیابی اثر کف پا با استفاده از شاخص قوس کف پاي استاهلی، شاخص قوس (AI یا  Arch Index) آنها تعیین شد. با استفاده از روشهاي آمار توصيفي، ميانگين و شاخص قوس با نرم افزار SPSS  نسخه 18براي سنین و جنسهای مورد نظر با فاصله اطمینان 95٪ بدست آمد. یافته‌ها: شاخص AI برای پسران و دختران 9 تا 14 ساله اصفهاني بترتيب (22/0 79/0) و (23/0 72/0) ، پسران و دختران 15 تا 24 ساله (19/0 75/0) و (18/0 71/0)، مردان و زنان 25 تا44 ساله (13/0 73/0) و (17/0 70/0)، مردان و زنان 45 تا 64 ساله (10/0 72/0) و (16/0 73/0)، مردان و زنان 65 سال و بالاتر (11/0 72/0)  و (18/0 77/0) بود. نتیجه­گیری: با توجه به نتایج این تحقیق، شاخص قوس پای زنان و مردان تعیین شد. همچنین در گروه سنی زیر 14 سال پسران و بالاي 65 سال زنان؛ بالاترين مقدار ميانگين AI مشاهده شد. بدون توجه به سن، ميانگين AI مردان كمي بالاتر از زنان به دست آمد ( ولی از نظر آماري معني‌دار نبود ( 05/0 p<) ).

Title: شیوع اختلال هماهنگی رشدی در کودکان 3 تا 11 ساله ایرانی

http://jrrs.mui.ac.ir/index.php/jrrs/article/view/1255

چکیده مقدمه: اختلال هماهنگي رشدي يک ناتواني دراز مدت بوده و مشکلات  اجتماعي، عاطفي و تحصيلي مرتبط با آن ممکن است تا دوران نوجواني و بزرگسالي ادامه پيدا کنند. به همين دليل نياز به شناسايي و مداخله‌هاي اوليه در مورد اين کودکان ضروري به نظر مي‌رسد. در این راستا هدف از این مطالعه برآورد میزان شیوع اختلال هماهنگی رشدی در کودکان 3 تا 11 ساله ایرانی بود.    مواد و روش‌ها:  تحقیق حاضر از نوع توصیفی و شیوه انجام آن زمینه‌یابی بود. به‌طوریکه از روش نمونه‌گیری چند مرحله‌ای سیستماتیک (منظم) برای انتخاب نمونه تحت بررسی استفاده شد. در مجموع والدین 1784 پرسشنامه اختلال هماهنگی رشدی را تکمیل کردند. مطالعه با 15 درصد کودکانی که پایین‌ترین نمرات کل را از نظر والدینشان دریافت کرده بودند ادامه یافت. به منظور ارزیابی عملکرد حرکتی کودکان و برآورد میزان شیوع اختلال هماهنگی رشدی از آزمون رشد حرکتی درشت-2 استفاده شد. کودکانی که هر گونه مشکل پزشکی شناخته شده داشتند یا بهره هوشی آنها کمتر از 70 بود از روند مطالعه کنار گذاشته شدند. از آزمون آماری خی دو در سطح معناداری 05/0 = α برای تجزیه و تحلیل داده‌ها استفاده شد. یافته‌ها: شیوع اختلال هماهنگی رشدی در نمونه تحت بررسی 7/2 درصد بود. به‌طوریکه این میزان در پسران 53/3 درصد و در دختران 85/1 درصد و نسبت جنسیت 2 به 1 بود. نتیجه‌گیری: با وجود اینکه این میزان کمتر از گزارش‌های مطالعات انجام شده قبلی می‌باشد، توجه بیش از پیش والدین و مسئولین نسبت به اختلال هماهنگی رشدی، غنی‌تر کردن برنامه‌های کلاسی تربیت‌بدنی و ترغیب کودکان به سمت فعالیت‌های حرکتی به منظور پیشگیری از مشکلات ثانویه مرتبط با این اختلال توصیه می‌شود.

Title: رابطۀ رشد زبان با پرخاشگری جسماني در کودکان کم‌‌ توانی ذهني آموزش‌پذیر

http://jrrs.mui.ac.ir/index.php/jrrs/article/view/1250

چکیده مقدمه: یکی از رایج­ترین مشکلات رفتاری در کودکان کم­توان ذهنی، پرخاشگری جسمانی است. پرخاشگری جسمانی به رفتارهایی مانند کتک­زدن، لگدزدن و پرتاب­کردن اشیاء و تهدید به انجام این فعالیت­ها اشاره دارد که همگی به نیت صدمه­زدن به دیگران صورت می­گیرد. پرخاشگری جسمانی با عوامل مختلفی از جمله مهارت­های زبانی در ارتباط است که در این مطالعه به بررسی آن در کودکان کم­توان ذهنی پرداخته شده است. مواد و روش‌ها: تحقیق حاضر به روش غیرتجربی و مقطعی انجام شد. 102کودک کم­توان ذهنی آموزش­پذیر (51 پسر و 51 دختر)  6 تا 8 ساله که معیارهای ورود به مطالعه را داشتند، به روش نمونه­گیری تصادفی طبقه­ای (سن و جنسیت) از مراکز آموزشی کودکان کم­توان ذهنی شهر شیراز انتخاب و با استفاده از نسخۀ فارسی آزمون رشد زبان (TOLD-P3) و پرسشنامۀ پرخاشگری آشکار و رابطه­ای، ارزيابی شدند. به ­منظور بررسی میزان همبستگی بین مهارت­های زبانی (زبان بیانی و زبان درکی) و پرخاشگری جسمانی در کودکان کم­توان ذهنی از ضریب همبستگی پیرسون و جهت مقایسۀ پرخاشگری جسمانی دختران و پسران کم­توان ذهنی از آزمون تی ­ـ مستقل در سطح معنی­داری 05/0 استفاده شد. یافته­ها: بین پرخاشگری جسمانی و مهارت زبان بیانی (008/0=p) و زبان درکی (019/0=p) رابطۀ منفی و معنی­دار وجود داشت. به عبارت دیگر، با کاهش مهارت­های زبان بیانی و زبان درکی در کودکان کم­توان ذهنی احتمال بروز پرخاشگری جسمانی در آن‌ها وجود دارد. میزان بروز پرخاشگری جسمانی در  پسران به ­طور معنی­داری بیشتر از دختران بود (014/0=p). همچنین، تفاوت معنی‌داری بین مهارت زبان بیانی و زبان درکی دختران و پسران کم­توان ذهنی وجود نداشت. نتیجه­گیری: بین مهارت زبان بیانی، زبان درکی و پرخاشگری جسمانی همبستگی منفی و معنی­دار وجود دارد. علاوه­بر این، مهارت­های زبان بیانی ضعیف در دختران کم­توان ذهنی باعث بروز رفتار پرخاشگرانه جسمانی در آن‌ها می­شود. در حالی­که، نمی­توان رابطه­ای بین مهارت­های زبانی (زبان بیانی و زبان درکی) و پرخاشگری جسمانی پسران کم­­توان ذهنی قائل شد.

Title: بررسی عوامل تسهیل‌کننده و بازدارنده یادگیری مهارت‌های عملی از دیدگاه دانشجویان سال سوم به بعد رشته‌های توانبخشی

http://jrrs.mui.ac.ir/index.php/jrrs/article/view/1245

چکیده مقدمه: این مطالعه با هدف تعیین عوامل تسهیل‌کننده و بازدارنده یادگیری مهارت‌های عملی از دیدگاه دانشجویان سال سوم به بعد رشته‌های توانبخشی دانشگاه علوم پزشکی اصفهان انجام شد. مواد و روش‌ها: این مطالعه توصیفی از نوع مقطعی می‌باشد که کلیه دانشجویان سال سوم به بعد رشته‌های ارتوپدی فنی، فیزیوتراپی، گفتاردرمانی و کاردرمانی جهت ورود به مطالعه انتخاب شدند و پرسش‌نامه محقق‌ساخته که حاوی 62 سؤال در خصوص عوامل تسهیل‌کننده و بازدارنده مؤثر بر یادگیری مهارت‌های عملی بود استفاده گردید. سؤالات  به 8 حيطه تقسيم و بر مبناي مقياس ليكرت و از کاملاً موافقم= 5 تا کاملاً مخالفم = 1 امتیازبندی گرديد.  داده‌ها در نرم‌افزار spss  توسط آزمون‌هاي توصیفی و تحلیل واریانس با اندازه‌گیری‌های مکرر مورد تجزیه و تحلیل قرار گرفت. یافته‌ها: 100 دانشجوی سال سوم به بعد با میانگین سنی   8/.± 2/22 سال مورد مطالعه قرار گرفتند. در حيطه عوامل تسهیل‌کننده آموزش مهارت‌هاي عملي، دانشجويان بيشترين موافقت (انتخاب گزینه کاملاً موافقم و موافقم) را با حیطه ویژگی‌های تسهیل‌کننده مربیان (85%)  و ارتباط بین مربی و دانشجو (85%) بيان نمودند. در حيطه عوامل بازدارنده، دانشجويان بيشترين موافقت را با حیطه امکانات  ضعیف موجود بیمارستانی و دانشگاهی (76%) بيان نمودند. نتیجه‌گیری: مهم‌ترين حیطه تسهيل‌کننده آموزش باليني از ديدگاه دانشجويان، ویژگی‌ها و معیارهای خوب مربیان بود. لذا برنامه‌ریزی در جهت بکارگیری اساتید مجرب و آموزش‌های مداوم  مربیان کم تجربه توصيه مي‌گردد. مهم‌ترين حیطه بازدارنده آموزش باليني ، عدم امکانات لازم در بخش‌ها و كلينيك‌ها بود. در نتیجه چنان‌چه کمبود امکانات و تجهیزات لازم در محیط بالینی را با یک برنامه‌ریزی مناسب جبران نماييم در كارآمد كردن دانشجويان تأثير بسزایی خواهد داشت.

Title: بررسی افت ناوی و تورشن تیبیا در دانشجویان دختر فعال مبتلا به سندرم درد پاتلوفمورال و سالم

http://jrrs.mui.ac.ir/index.php/jrrs/article/view/1044

چکیده مقدمه: سندرم درد پاتلوفمورال مشكلی بسيارشايع درد زانو مي‌باشد كه 20 تا 40 درصد دردهاي زانو و 10 تا 20 درصد بيماران كلينيك‌هاي فيزيوتراپي را تشكيل مي‌دهد. هدف از این تحقیق، بررسی افت ناوی وتورشن تیبیا دردانشجویان دختر جوان فعال مبتلا به سندرم درد پاتلوفمورال وسالم می‌باشد. مواد و روش‌ها: در این پژوهش 30 دانشجوی دختر فعال با دامنه سنی 20 تا 30 سال به صورت در دسترس هدفمند به عنوان نمونه آماری انتخاب شده و به دو گروه مبتلا به سندرم پاتلوفمورال (15 نفر) و سالم (15 نفر) تقسیم شدند. به منظور اندازه‌گیری میزان تورشن تیبیا، افت ناوی و شدت درد به ترتیب از گونیامتر، خط کش مندرج و مقیاس دیداری درد (Visual Analog Scale) VASاستفاده شد. برای تجزیه وتحلیل آماری داده‌ها از آزمون tمستقل در سطح معنی‌داری استفاده گردید(05/0 ≥P). همچنین از آزمون (Interclass Correlation Coefficient) ICC برای بررسی تکرار پذیری داده‌های اندازه‌گیری شده، استفاده شد. یافته‌ها: با توجه به یافته‌های پژوهش اختلاف معناداری بین متغیرهای افت ناوی و تورشن تیبیا در دو گروه سالم و مبتلا به سندرم پاتلوفمورال مشاهده نگردید. نتیجه‌گیری: با توجه به نتایج پژوهش حاضر، بهنظرمی‌رسدکه افت ناوی وتورشن تیبیا متناسب باویژگی‌های فردی وسابقه تمرینی افراد متغیر است و ممکن است ریسک فاکتوری برای سندرم پاتلوفمورال درافراد فعال نباشد.

Title: تأثیر ارتز گوه خارجی بر بالانس استاتیک افراد مبتلا به استئوآرتریت در ناحیه داخلی زانو

http://jrrs.mui.ac.ir/index.php/jrrs/article/view/1626

چکیده مقدمه: استئوآرتریت بخش داخلی زانو یکی از شایع‌ترین دلایل ناتوانی و محدودکننده فعالیت در بزرگسالی می‌باشد. اختلالات تعادلی در این افراد گزارش شده است. ارتزهای گوه خارجی می‌تواند تأثیرات فوری و طولانی مدتی را بر زانو داشته باشند که در نتیجه ممکن است تأثیرات مهمی بر تعادل این افراد داشته باشند. بنابراین هدف از این مطالعه بررسی تأثیر چهار نوع ارتز گوه خارجی بربالانس استاتیک افراد مبتلا به استئوآرتریت در ناحیه داخلی زانو می‌باشد. مواد و روش‌ها: 18 زن مبتلا به استئوآرتریت در ناحیه داخلی زانو مورد مطالعه قرار گرفتند. بالانس استاتیک طی ایستادن روی دو اندام با چشمان باز و با پوشیدن کفش یکسان با استفاده از دستگاه سکوی نیرو و با پوشیدن پنج نوع گوه خارجی ( 5/8 درجه عرضی) در داخل و خارج کفش بوسیله ی پارامترهای مرتبط با  مرکز فشار ارزیابی شد. یافته‌ها: استفاده از گوه‌های خارجی تأثیر معنی‌داری بر متغیرهای بالانس استاتیک در مقایسه با کفی ساده نداشت (05/0P>)، اگرچه استفاده از ارتز گوه خارجی منجر به مقادیر پایین‌تری در میانگین متغیرهای اندازه‌گیری شده، نسبت به کفی ساده گردید. بعلاوه مقایسه بین چهار نوع گوه خارجی نشان داد که گوه‌های خارجی در خارج کفش در مقایسه با گوه خارجی پاشنه در داخل کفش  منجر به کاهش معنی‌دار مقادیر میانگین برآیند متوسط مسافت طی شده  مرکز فشار و مساحت ناحیه بیضی و دایره محاط بر محدوده حرکت مرکز فشار گردیدند (05/0P<) نتیجه گیری: هر چند استفاده از گوه‌های خارجی تأثیر معنی‌داری در پارامترهای بالانس افراد مبتلا به استئوارتریت در وجه داخلی زانو نداشت، اما در مقایسه با کفی ساده، استفاده از ارتز گوه خارجی منجر به مقادیر پایین‌تری در پارامترهای بالانس این افراد گردید که شاید نشانگر بهبود تعادل با استفاده از گوه‌های خارجی باشد. در مقایسه انواع گوه‌های خارجی، ارتز گوه خارجی در خارج از کفش منجر به بهبود بیشتر بالانس گردید.

Title: مروری نظام‌مند بر مطالعات انجام گرفته در زمینه اسب‌درمانی (Hippotherapy) و گزارش توصیفی نتایج آن‌ها

http://jrrs.mui.ac.ir/index.php/jrrs/article/view/1076

چکیده مقدمه: اسب درمانی یکی از روشهای توانبخشی میباشد که در سالهای اخیر کاربرد زیادی یافته است. هدف مطالعه حاضر جمع بندی مطالعات و گزارش توصیفی نتایج اسب درمانی در درمان و توانبخشی ناتوانی ها و معلولیت ها میباشد. مواد و روش‌ها: این مطالعه از نوع مطالعات مروری نظام‌مند می‌باشد. اطلاعات مورد نیاز از طریق جستجو در پایگاه‌های داده‌ای: PubMed ، Medline، ,Cinahl , Web of knowledge , Science Directو SIDبا استفاده از کلیدواژه‌های hippotherapy، Equine- assisted therapy, Horseback riding, Horse riding , Therapeutic riding, و اسب درمانی جمع‌آوری گردید. بازه زمانی جمع‌آوری مقالات بین سال‌های 2012 – 1990 انتخاب گردید و در نهایت تعداد 35 مقاله وارد مطالعه گردید. مقالات انتخاب شده به طور کامل مورد مطالعه قرار گرفته و نتایج با استفاده از (Extraction table) استخراج، خلاصه‌سازی و گزارش گردید. یافته‌ها: میانگین تعداد نمونه‌ها برای هر مطالعه برابر با 5/22 نفر بود. میانگین مدت زمان مداخله برای هر مطالعه نیز برابر با 16/13 هفته بود. بیشترین گروه سنی مورد مطالعه کودکان بودند و بیشترین ناتوانی بررسی شده نیز فلج نخاعی بود. در 33 مطالعه (حدود94%) نتایج مطالعات نشان دهنده تاثیر مثبت و اثربخش اسب درمانی بود. نتیجه‌گیری: بر اساس نتایج مطالعات بررسی شده اسب درمانی تاثیر مثبتی در توانبخشی داشته و این اثربخشی در مواردی مانند فلج نخاعی، ثبات سر و اندام‌های فوقانی، مالتیپل اسکلروسیز، ناتوانی‌های فیزیکی و ... مشاهده می‌شود. با این وجود مطالعات صورت گرفته در این زمینه محدود بوده و نمی‌توان با این تعداد نتیجه‌گیری کرد و نیاز به انجام مطالعات بیشتر در این زمینه می‌باشد.

Title: مروری بر کاربردهای سیستم BCI در علم توان‌بخشی با رویکرد بازتوانی ارتباط با محیط پیرامون

http://jrrs.mui.ac.ir/index.php/jrrs/article/view/1075

چکیده   مقدمه: مشکلاتی که پس از ضربه مغزی یا بیماری‌های دستگاه عصبی رخ می‌دهد منجر به بروز محدودیت‌های حرکتی و کلامی برای مدت طولانی در افراد می‌شود. پیشرفت‌های صورت گرفته در زمینه ارتباط مغز انسان و کامپیوتر (BCI) امکان شناسایی و طبقه‌بندی فعالیت‌های الکتریکی و متابولیک مغز و تبدیل آنها به یک فرمان کنترلی برای کامپیوتر و یا یک دستگاه مخصوص را فراهم می‌نماید. هدف از مطالعه حاضر مروری بر کاربردهای سیستم BCI در علم توان‌بخشی با رویکرد بازتوانی ارتباط با محیط پیرامون بود. مواد و روش‌ها: هدف استفاده از سیستم BCI به طور کلی یا ایجاد یک توانایی از دست رفته در فرد و یا بهبود توانایی تحلیل رفته می‌باشد. بر همین اساس سه کاربرد عمده برای سیستم BCI عبارت است از ایجاد امکان حرکت اعضای بدن، ایجاد قدرت تکلم و کنترل تجهیزات مختلف به منظور انجام فعالیت‌های روزانه. برای بررسی پیشرفت‌های صورت گرفته در این عرصه‌ها مقالات مرتبط با موضوع که تاکنون در مجلات و کنفرانس‌های معتبر ارایه گردیده مطالعه شده است. یافته‌ها: مفاهیم و اصول BCI به همراه فن‌آوری‌های مورد استفاده در این عرصه و آخرین پیشرفت‌ها در زمینه بهبود عملکرد سیستم‌های BCI در این مقاله ارایه شده است. در انتها نیز ظرفیت‌های موجود برای استفاده از سیستم  BCIو کارهای مورد نیاز برای توسعه بیشتر استفاده از آن در توان‌بخشی مورد بحث قرار گرفته است. نتیجه‌گیری: در 20 سال اخیر تلاش‌های بسیاری به منظور افزایش راندمان و سرعت انتقال داده در سیستم های BCI مبتنی بر EEG صورت گرفته است. برای دستیابی به این اهداف یک سیستم تبادل داده پرسرعت بین مغز و کامپیوتر مورد نیاز می‌باشد. تاکنون راهکارهای گوناگونی برای ایجاد یک ارتباط بدون تاخیر بین دستورات مغزی صادر شده و فرامین کنترلی تولید شده برای دستگاه‌ها معرفی شده است.

Title: بررسی تأثیر سیلیکون لاینر بر تعادل ایستگاهی یک فرد آمپوته زیر زانو: گزارش موردی

http://jrrs.mui.ac.ir/index.php/jrrs/article/view/1294

چکیده مقدمه: قطع عضو اندام تحتانی به علت تروما، بیماری‌های عروقی، دیابت و اختلالات مادرزادی ایجاد می‌شود.آمپوته‌ها توانایی راه رفتن و ایستادن را بر طبق سطح قطع عضوشان از دست می‌دهند. انواع مختلفی از پروتزها جهت افزایش کارایی آمپوته مورد استفاده قرار می‌گیرد. تأثیر نوع سیستم تعلیق پروتز بر اختلالات بالانس به طور شفاف مشخص نشده است. بنابراین هدف از این مقاله یافتن ارتباط بین تعادل ایستگاهی افراد آمپوته زیر زانو و نوع سیستم تعلیق می‌باشد. مواد و روش‌ها: در این مطالعه یک مرد 48 ساله با قطع عضو زیر زانوی یکطرفه بدون هیچ مشکل اسکلتی عضلانی شرکت داده شد. یک صفحه نیروی کیستلر با مبدل‌های پیزوالکتریک نیرو برای اندازه‌گیری پارامترهای مرکز فشار بدن مورد استفاده قرار گرفت. نوسان فرد در صفحه قدامی خلفی و داخلی-خارجی در حین استفاده از دو نوع پروتز (پروتز با سوکت سنتی و پروتز با سوکت سیلیکون لاینر) مورد ارزیابی قرار گرفت. یافته‌ها: میانگین نوسان مرکز فشار در حین استفاده از پروتز سنتی در صفحه قدامی-خلفی و داخلی خارجی به ترتیب برابر 093/23 (با خطای استاندارد: 14/4) و 53/22 (با خطای استاندارد: 02/5) در حالت چشم باز می‌باشد در حالی که این اعداد برای پروتز سیلیکون لاینر در صفحه قدامی-خلفی و داخلی-خارجی به ترتیب برابر با 35/21 (با خطای استاندارد: 30/4 ) و 5/15 (با خطای استاندارد : 56/4 ) است. میانگین نوسان مرکز فشار در جهت قدامی-خلفی و داخلی-خارجی در حین استفاده از پروتز با سوکت فومی بیش‌تر از سوکت سیلیکونی است (p value=0.03) نتیجه‌گیری: این مطالعه نشان داد کارایی آمپوته در حین ایستادن با سیلیکون لاینر به علت افزایش حس عمقی و تحریک رسپتورهای سطحی پوست بیش از نوع دیگر است. در حقیقت علاوه بر مزایای متعدد سیستم‌های سیلیکون لاینر افزایش بالانس و حس عمقی را می‌توان به این مزایا اضافه نمود.
